# The daily dynamics of cystic fibrosis airway microbiota during clinical stability and at exacerbation

**DOI:** 10.1186/s40168-015-0074-9

**Published:** 2015-04-01

**Authors:** Lisa A Carmody, Jiangchao Zhao, Linda M Kalikin, William LeBar, Richard H Simon, Arvind Venkataraman, Thomas M Schmidt, Zaid Abdo, Patrick D Schloss, John J LiPuma

**Affiliations:** Department of Pediatrics and Communicable Diseases, University of Michigan Medical School, Ann Arbor, MI 48109 USA; Department of Pathology, University of Michigan Medical School, Ann Arbor, MI 48109 USA; Department of Internal Medicine, University of Michigan Medical School, Ann Arbor, MI 48109 USA; Department of Microbiology and Immunology, University of Michigan Medical School, Ann Arbor, MI 48109 USA; USDA-ARS, South Atlantic Area, Athens, GA USA

**Keywords:** Cystic fibrosis, Respiratory exacerbation, Lung microbiome, Airway microbiome

## Abstract

**Background:**

Recent work indicates that the airways of persons with cystic fibrosis (CF) typically harbor complex bacterial communities. However, the day-to-day stability of these communities is unknown. Further, airway community dynamics during the days corresponding to the onset of symptoms of respiratory exacerbation have not been studied.

**Results:**

Using 16S rRNA amplicon sequencing of 95 daily sputum specimens collected from four adults with CF, we observed varying degrees of day-to-day stability in airway bacterial community structures during periods of clinical stability. Differences were observed between study subjects with respect to the degree of community changes at the onset of exacerbation. Decreases in the relative abundance of dominant taxa were observed in three subjects at exacerbation. We observed no relationship between total bacterial load and clinical status and detected no viruses by multiplex PCR.

**Conclusion:**

CF airway microbial communities are relatively stable during periods of clinical stability. Changes in microbial community structure are associated with some, but not all, pulmonary exacerbations, supporting previous observations suggesting that distinct types of exacerbations occur in CF. Decreased abundance of species that are dominant at baseline suggests a role for less abundant taxa in some exacerbations. Daily sampling revealed patterns of change in microbial community structures that may prove useful in the prediction and management of CF pulmonary exacerbations.

**Electronic supplementary material:**

The online version of this article (doi:10.1186/s40168-015-0074-9) contains supplementary material, which is available to authorized users.

## Background

Chronic pulmonary infection and inflammation culminating in respiratory failure is the primary cause of death in people with cystic fibrosis (CF). Studies employing culture-independent analyses have shown that CF airways typically harbor complex microbial communities comprised of numerous bacterial species, particularly during the early and intermediate stages of lung disease [1-9]. While these studies have made important contributions toward understanding the dynamics of these communities, they have been limited by cross-sectional design and/or their reliance on analysis of a very small number (often only one or a few) of respiratory samples taken during periods of interest. Samples representing the periodic exacerbations of respiratory symptoms that characterize CF most often have been obtained *after* the initiation of antibiotics for exacerbation treatment. A more complete understanding of the dynamics of airway microbiota, in particular with respect to changes occurring at the time of pulmonary exacerbation, would benefit from an analysis of serial samples obtained in the days *preceding* and coincident with exacerbation onset, but prior to the initiation of antibiotic therapy for exacerbation. A meaningful assessment of airway microbial community changes during this crucial period can only be made, however, in the context of an understanding of the day-to-day variability of airway communities during periods of clinical stability. Rigorous analyses of the daily dynamics of the airway microbiota around these critical periods are lacking.

We obtained daily sputum samples from persons with CF in order to characterize changes in the bacterial and viral airway community during periods of clinical stability and during the onset of exacerbation of clinical symptoms, but prior to the initiation of antibiotics for exacerbation. We quantified community stability to assess the day-to-day variation in community structures and examine the degree of inter-individual variation in microbiome stability. We also describe the changes in community structure at the onset of symptoms of exacerbation in these four individuals.

## Results

We analyzed the bacterial microbiota in sputum samples obtained daily from four individuals, referred to as subjects A, B, C, and D (Table 1), during approximately 25-day intervals that included a period of clinical stability (‘baseline’) followed by the onset of symptoms that eventually led to the prescription of antibiotics for treatment of pulmonary exacerbation. In total, 95 samples from these four subjects were analyzed, plus six control samples (see Methods), which were sequenced in duplicate (‘replicate controls’) to assess the degree of variability inherent in the methodology.

**Table 1:** **Demographic and sample collection information for four study subjects**

**Subject**	**Age**	**Gender**	**CFTR mutation**	**%** **FEV**_**1**_^**a**^	**Most abundant OTU at baseline**	**Number of samples**
A	46	F	ΔF508/N1303K	68	*Staphylococcus*	20
B	31	F	ΔF508/not identified	75	*Burkholderia*	27
C	37	F	ΔF508/ΔF508	88	*Streptococcus*	26
D	20	F	ΔF508/ΔF508	55	*Burkholderia*	22

^a^Most recent measurement of percent predicted forced expiratory volume in 1 s. Patient A, 26 days prior to sample day 1; patient B, sample day 27; patient C, 7 days prior to sample day 1; patient D, sample day 14.

### Day-to-day community variation when clinically stable

**Figure 1:**
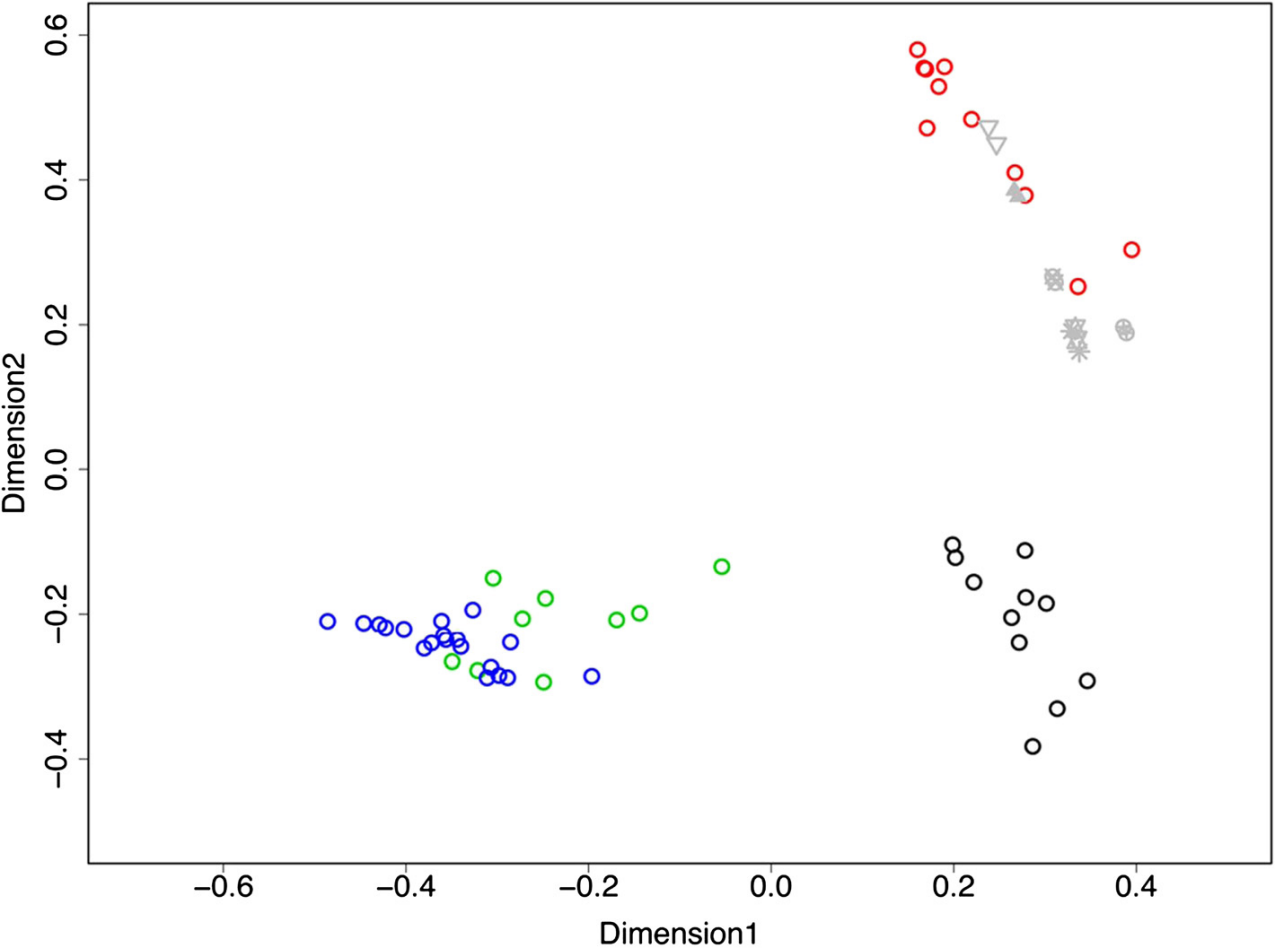
**Baseline community structure.** Bray-Curtis-based nonmetric multidimensional scaling plot showing daily sputum samples from four study subjects (subject A is red; subject B is green; subject C is black; subject D is blue), collected during clinically stable periods. Pairs of other symbols in gray are same sample replicate controls from subject A as described in Methods.

The baseline samples from each subject separated into clusters in a Bray-Curtis (BC)-based nonmetric multidimensional scaling (NMDS) ordination plot (Figure 1). Samples separated by dominant genera with samples from subjects B and D occupying a similar space on the ordination plot since their communities were both dominated by *Burkholderia*. Samples from subject A (*Staphylococcus* dominant) and subject C (*Streptococcus* dominant) clustered into different spaces. The replicate control samples analyzed on the same DNA sequencing run clustered tightly, reflecting the low degree of variability in community measures among these samples.

**Figure 2:**
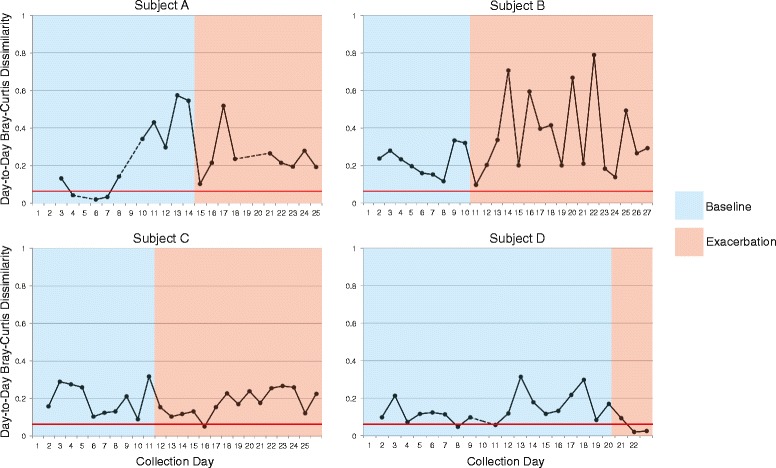
**Daily change in community structure from baseline into exacerbation.** Day-to-day changes in airway bacterial community structure in four persons with CF. Bray-Curtis dissimilarity between consecutive daily samples is shown during periods of clinical stability (blue) that ended with the onset of symptoms of exacerbation (pink). Dashed lines indicate more than one day between samples. Red horizontal line indicates the maximum Bray-Curtis dissimilarity between replicate control samples. Each plot ends on the day preceding the prescription of antibiotics for treatment of exacerbation.

For each subject, the average pairwise BC dissimilarity observed between baseline samples was greater than that found between the replicate control samples (red line in Figure 2). Each subject had higher median BC dissimilarity among all pairwise baseline samples compared to the replicate control samples, indicating that the community variability between baseline samples exceeded what would be expected due to background methodological ‘noise’.

We next asked whether the degree of day-to-day change in baseline bacterial community structure varied between subjects and/or within subjects over time. The mean BC dissimilarity among day-to-day pairwise comparisons of all baseline samples varied between the four subjects, with subject A having the highest mean (0.255) and subject D having the lowest (0.144) (Figure 2). The mean BC dissimilarity during the second week of baseline samples from subject A was greater than that observed during the prior week (0.388 vs. 0.057, respectively). Further, a very strong negative correlation was observed between day-to-day BC dissimilarity and days to start of symptoms for subject A, indicating that the community became more variable as this subject approached exacerbation (*P* = −0.842). In contrast, strong correlations between day-to-day BC dissimilarity and days to the start of symptoms were not observed for the other three subjects (*P* < 0.6).

Homogeneity of molecular variance (HOMOVA), a nonparametric analog to Bartlett's test for homogeneity of variance, was used to determine if subjects differed significantly from each other with respect to overall variability in community structure at baseline. Significantly greater variation in community structure was observed in subject A, compared to either subject C or subject D (*P* = 0.002 and 0.003, respectively), but not in comparison to subject B (*P* = 0.452). In contrast, no statistically significant differences were observed in the degree of variation in community structure at baseline between subjects B, C, and D.

### Community movement during exacerbation

**Figure 3:**
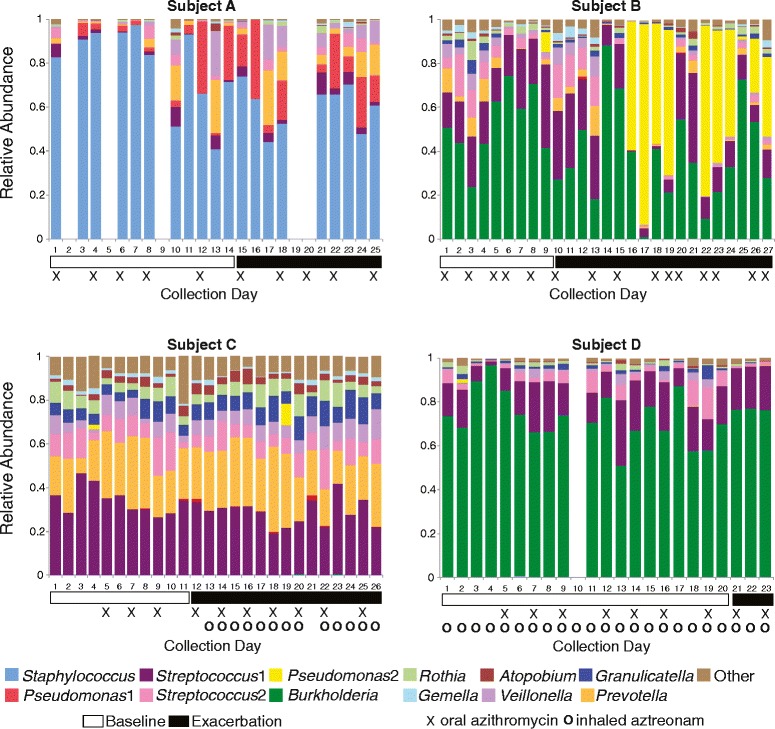
**Relative abundance of top OTUs in daily samples.** Relative abundance of the top OTUs in consecutive daily sputum samples collected from four subjects during periods of clinical stability (white horizontal bars) and onset of exacerbation (black horizontal bars). Symbols below plots indicate days when maintenance antibiotics were taken. Each plot ends on the day preceding the prescription of antibiotics for treatment of exacerbation.

**Figure 4:**
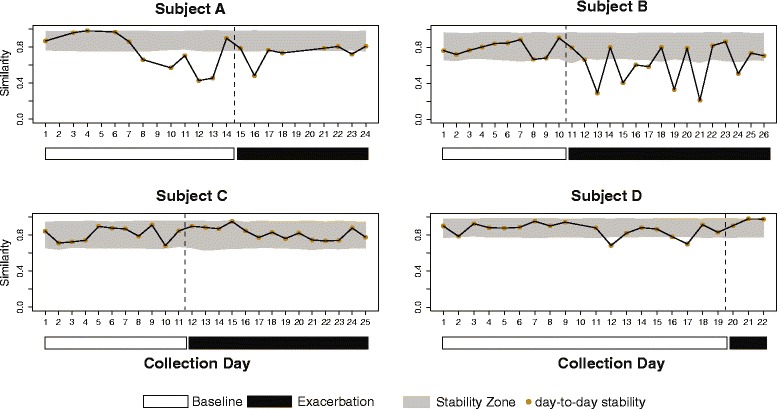
**Community stability around exacerbation.** Points represent the Bray-Curtis similarity (1 − Bray-Curtis dissimilarity) between the relative abundances of all OTUs at any given time point and the next time point, and range from 0 (no stability) to 1 (perfect stability). (See Methods for details.) The gray-shaded regions represent the ‘stability zone,’ which is the expected range of similarity for each subject. Periods of clinical stability (white horizontal bars) and onset of exacerbation symptoms (black horizontal bars) are indicated. Dashed vertical lines indicate transition between baseline and exacerbation. Each plot ends on the day preceding the prescription of antibiotics for treatment of exacerbation.

Similar analyses of day-to-day changes in community structure during onset of exacerbation symptoms (*prior to antibiotic treatment*) revealed different patterns of change between subjects as clinical symptoms developed, suggesting different types of exacerbations, some of which were correlated with substantial shifts in airway bacterial communities. Communities in subject A showed marked changes in the week prior to the onset of exacerbation symptoms (Figures 2, 3, and 4 and Additional file 1: Table S1). Communities in subject B showed marked change just after the onset of symptoms. The communities in subject C and subject D remained relatively unchanged with onset of exacerbation symptoms.

Study subjects recorded symptoms on a daily questionnaire based on the Respiratory Symptoms Domain of the Cystic Fibrosis Questionnaire-Revised [10,11], and a symptom score was calculated by summing the total number of positive symptoms each day. We observed no strong correlations between day-to-day BC dissimilarity and either symptom score or change in symptom score from the previous day (*P* < 0.6). When grouped into baseline and exacerbation categories, the average day-to-day BC dissimilarity was higher for exacerbation samples compared to baseline samples for subject B (0.361 and 0.213, respectively), and lower for exacerbation samples compared to baseline samples for subject D (0.047 and 0.144, respectively). (It should be noted, however, that only three exacerbation samples were available for subject D prior to the initiation of antibiotics). No large differences in average day-to-day BC dissimilarity were observed when comparing day-to-day BC dissimilarity of exacerbation samples to baseline samples for subject A (0.246 and 0.255, respectively) and subject C (0.177 and 0.196, respectively).

**Figure 5:**
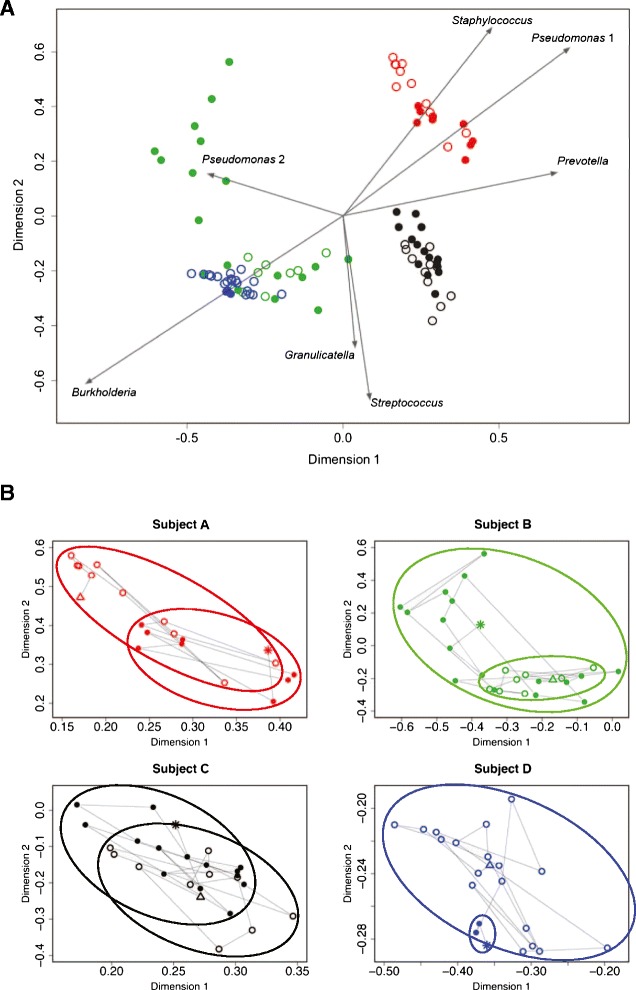
**Community movement before and during exacerbation.** Bray-Curtis-based nonmetric multidimensional scaling plots showing daily sputum samples from four subjects (subject A is red; subject B is green; subject C is black; subject D is blue) collected during periods of clinical stability (open circles) and at onset of symptoms of exacerbation (closed circles). **A** Arrows indicate relative influence of the specified OTUs on the position of samples in the ordination space. **B** Each subject's plot is shown separately with lines connecting samples from first (open triangle) to last (star) collected (stress = 0.151). Each subject's plot is magnified to fill the ordination space and highlight movement between that subject's samples. Baseline samples are enclosed by a dashed ellipse; exacerbation samples are enclosed by a solid ellipse.

While the communities in subject A's samples showed an increase in both Shannon diversity (*P* = 0.614) and evenness (*P* = 0.653) as this subject approached antibiotic therapy, no strong trends in alpha diversity over time were observed in the other three subjects (*P* < 0.6) (Additional file 2: Figure S1). Changes in community structures at exacerbation were visualized on BC-based NMDS plots (Figure 5).

After exploring global community changes, we looked for patterns of change in specific genera at exacerbation onset. A strong negative correlation was observed between collection day and the relative abundance of *Staphylococcus* for subject A (*P* = −0.659), while a moderate correlation was observed between collection day and the relative abundance of *Streptococcus* for subject C (*P* = −0.426). The average relative abundance of these operational taxonomic units (OTUs) was lower in exacerbation samples compared to baseline samples in both subjects (A 0.607 vs. 0.788, respectively; C 0.290 vs. 0.342, respectively). The relative abundance of the dominant OTU for subject B (*Burkholderia*) also decreased at exacerbation compared to baseline (average relative abundance 0.525 vs. 0.387, respectively), although we observed only a weak negative correlation between collection day and *Burkholderia* relative abundance (*P* = −0.253). During exacerbation, the community structure of subject B's samples was quite variable; large day-to-day shifts in relative abundances of the top OTUs were observed (Figure 3, Additional file 1: Table S1). No large changes in relative abundance of subject D's dominant OTU were observed at exacerbation, nor were strong correlations observed between absolute abundance of the dominant OTUs (calculated as the product of total bacterial load and the relative abundance of the OTU) and collection day for any subject.

Total bacterial load was not markedly different between baseline and exacerbation samples for any of the four subjects, nor were strong correlations observed between bacterial load and symptom score (*P* < 0.6). While total bacterial load for subjects B, C, and D fluctuated within a 2-log range over the entire period of observation, the total load for subject A was more variable, fluctuating over a greater than 3-log range (Additional file 3: Figure S2). None of the 31 samples assessed for the presence of respiratory viruses tested positive by the FilmArray Respiratory Panel.

### Defining airway community stability

Bacterial community stability was quantified across the entire period of observation by defining, for each subject, a zone for changes in community structure, within which variations in community composition could be explained by stochastic events (‘stable’) as described in Methods (Figure 4). When communities move outside this zone, we interpret this to be due to a perturbation that changes the community structure (‘unstable’). Communities in subject A moved outside the stability zone 7 days *prior to* symptom onset, before becoming relatively stable again after 4 days of symptoms. Communities in subject B also moved outside the stability zone, but unlike subject A, this occurred *after* the onset of symptoms and continued until at least the beginning of antibiotic treatment. Communities in subject C remained within the stability zone for the entire period of observation, indicating relatively stable communities before and during exacerbation onset, while subject D moved outside the stability zone briefly in the week prior to the onset of symptoms.

## Discussion

Repeated exacerbations of pulmonary symptoms in persons with CF are associated with a progressive decline in lung function. Although clinical management based on antibiotic treatment and intensified airway clearance is usually at least partially effective [10-12], patients fail to return to 100% of baseline lung function in greater than 40% of exacerbations treated with intravenous antibiotics [13]. Unfortunately, relatively little is known about the pathophysiologic basis of exacerbations. The identification of microbial signals that correlate with exacerbation would aid efforts to prevent or mitigate the effects of these events and would inform studies investigating the mechanisms by which these factors may drive exacerbation. A prerequisite to understanding how CF airway communities change at exacerbation, however, is a better appreciation of the day-to-day variability in these communities during periods of clinical stability. Unfortunately, no previous studies have investigated CF airway microbial community variability with this level of granularity. To this end, we analyzed the daily dynamics of the airway bacterial communities in persons with CF during periods of clinical stability and at the onset of pulmonary exacerbations, prior to the initiation of antibiotic therapy for exacerbation.

In the absence of clinical symptoms, bacterial airway communities remained relatively stable. Subject A had overall greater variability at baseline than subjects C or D, due to destabilization of subject A's community during the week immediately preceding the onset of exacerbation symptoms. The airway community in subject D showed more subtle instability during this time. Because the baseline samples from all four subjects were obtained 1 to 3 weeks immediately preceding the onset of symptoms, it is possible that community variability was already influenced by impending exacerbation even in the absence of clinical symptoms. Analyses of daily samples obtained during periods of clinical stability further removed from exacerbation are warranted to better ascertain the degree of day-to-day variability in bacterial airway communities in CF in general. For the purposes of our study, however, we believed it is important to assess changes in community structure during the period of clinical stability immediately preceding the onset of exacerbation symptoms.

The consistency in these subject's airway communities over the course of several days has bearing on questions pertaining to bacterial biogeography within diseased lungs and on how well sputum samples represent airway communities in microbiome studies in CF. Recent studies have demonstrated that microbial communities vary over the spatial landscape of the diseased lung [14-17]. The remarkable level of community stability observed in our study subjects, particularly subject D over the first 10 days of observation (Figures 2 and 4), would not be expected if serial sputum specimens were sampling distinct regions of diseased lungs from 1 day to the next. This consistency also suggests that measures of lung microbial community structure derived from expectorated sputum are not substantially impacted by ‘contamination’ from oropharyngeal microbiota. In a recent comparison of multiple body sites, the temporal variability in oral cavity microbiomes was found to be significantly greater than those in vaginal, gut, skin, and anterior nares sites [18]. If sputum samples were significantly contaminated with oral microbiota during expectoration, we would expect to see variability reflecting that observed in serial oral cavity samples. Finer scale sampling of oral sites, analyzed alongside paired sputum samples from the same subject, would further illuminate this issue.

With the onset of symptoms of exacerbation, we observed distinct patterns of bacterial community change among the four subjects. The community in subject A showed considerable movement several days before the onset of symptoms, then shifted to an alternate stable state different in structure from the previous week. This change can be visualized by NMDS (Figure 5), wherein subject A's baseline samples occupy a different space in the ordination plot than the exacerbation samples, the result, in part, of a decrease in the relative abundance of *Staphylococcus* and increases in the relative abundances of *Pseudomonas* and *Prevotella* (Figure 3, Table S1).

The airway bacterial community in subject B destabilized coincident with the onset of exacerbation symptoms (Figure 4). However, in contrast to subject A, the community did not shift to a new structure (that is, the centroid of subject B's exacerbation samples in the NMDS plot remained near that of the baseline samples, Figure 5). However, approximately 1 week *after* the onset of symptoms, the community shifted intermittently from one dominated by *Burkholderia* to one dominated by *Pseudomonas*.

The communities in subjects C and D were relatively stable through the onset of symptoms (Figure 4). This result was unexpected for subject C, who had the greatest baseline community diversity among the subjects. Previous work suggested that a high degree of baseline community diversity predisposes to greater change at exacerbation [7]. Subject D was prescribed antibiotic therapy very soon (3 days) after the onset of exacerbation symptoms. The three daily samples immediately prior to antibiotic administration showed exceptionally stable communities, with a maximum BC dissimilarity between communities of only 0.032, which is within the range of the replicate controls. Whether more movement of this subject's airway community would have been observed in a larger set of samples before the initiation of treatment is unknown.

Despite the inter-subject differences in community dynamics with exacerbation onset in these four subjects, common features are also apparent. In three of four subjects, the dominant taxon (*Staphylococcus*, *Burkholderia*, and *Streptococcus* in subjects A, B, and C, respectively) *decreased* in relative abundance around the time of exacerbation. Although this change did not reflect a decrease in the absolute density of these species, due to day-to-day variability in total bacterial density, this finding is consistent with previous work that demonstrated decreases in the relative abundance of the dominant taxa at the time of exacerbation in another set of subjects [7]. The pathophysiologic implications of this intriguing finding require further elucidation, but suggest a role for less abundant taxa in some pulmonary exacerbations.

In all four subjects, despite subtle day-to-day variability in total bacterial load, we observed no overall changes in total bacterial density between baseline and exacerbation samples. This finding supports studies that similarly failed to show significant changes in overall bacterial density with exacerbation [3,7,9]. These findings suggest that shifts in the relative abundance of bacterial community members, rather than changes in total bacterial density, are more likely associated with alterations in clinical state.

Although respiratory viral infection is associated with a significant minority of pulmonary exacerbations in CF [19-21], we detected no respiratory viruses in samples obtained around the time of exacerbation onset in any of the four subjects. This analysis was limited to the 17 respiratory viruses included in the commercial viral detection system we employed. Nevertheless, this panel would be expected to detect the great majority of viruses responsible for viral respiratory infection in CF based on recent epidemiologic surveys [19,20].

Our study design allowed us to assess the correlation between subject-reported symptoms and airway community structure. In this regard, we observed no strong correlations between daily symptom scores and day-to-day community variability. However, it is possible that a larger scale study and/or a more refined symptom scoring system might detect such relationships, if they indeed exist. Weighting of symptoms, rather than merely summing the number of symptoms on each day, could reveal correlations with airway community movement that were not detected in this study. Similarly, consideration of other patient-specific features (for example, disease stage) and/or analysis of the temporal relationship between specific symptoms and microbiomic measures may prove worthwhile. Our study was limited to four subjects at a single center. Although samples from three of four subjects contained *Pseudomonas*, none were dominated by this taxon as is often seen in CF samples. The applicability of these findings to the larger CF population is thus unclear. Finally, we restricted our analyses to predominantly descriptive reports of trends within and between subjects, given the small sample size and correlated nature of the data. Our results indicate the potential for significance given increased power from an expanded pool of subjects.

Rapid advances in technical capability for microbiome analyses will provide opportunities to study larger sample sets to more completely characterize the spectrum of microbiome changes associated with pulmonary exacerbation. Transcriptomic and/or metabolomic analyses would complement these efforts in enhancing our understanding of how changes in the activity of airway microbiota relate to changes in the host's clinical state.

## Conclusion

Daily airway bacterial community structures in CF are relatively stable during periods of clinical stability. The onset of symptoms of exacerbation may be heralded by marked shifts in these communities, even in the absence of viral infection or antibiotic therapy for the treatment of exacerbation. Whether such shifts only occur in a minority of exacerbations, as observed here, is unclear. Monitoring of airway microbial community structures therefore has the potential to identify signatures that could be useful in predicting CF respiratory exacerbations, which, in turn, may enable more prompt and/or appropriate therapy of exacerbation. A better understanding of the changes in airway communities around the time of exacerbations will also inform studies to elucidate the pathophysiologic mechanisms of exacerbation in CF.

## Methods

### Sputum specimens and metadata

With approval of the Institutional Review Board of the University of Michigan Medical School, sputum specimens were collected daily by four persons with CF and stored at 4°C for up to 27 days before being sent on ice to the investigator's laboratory for processing and storage at −80°C. Subjects were part of a larger cohort of CF patients participating in long-term daily collection of sputum for microbiome analysis. The first four exacerbations captured from this cohort were chosen for analysis in this study. Study subjects recorded symptoms and antibiotic use on a daily questionnaire based on the Respiratory Symptoms Domain of the Cystic Fibrosis Questionnaire-Revised [22,23]. Symptoms recorded included increased cough, change in sputum, shortness of breath, fever, decreased energy, decreased appetite, and missed work/activities. Evidence of hemoptysis in the specimen was recorded by the laboratory. A symptom score was calculated by summing the total number of positive symptoms, plus hemoptysis, each day. Baseline samples were defined as those obtained during a period of consecutive symptom-free days that culminated in the onset of symptoms of exacerbation. ‘Exacerbation’ samples were defined as those obtained from the start of consistent symptoms to the day *prior to* the start of antibiotic treatment for exacerbation [7]. Six consecutive daily sputum samples obtained from subject A served as technical control samples. DNA was prepared from each of these six samples and each DNA samples was sequenced in duplicate on the same sequencing run. These six pairs of replicate control samples served to assess the degree of background variability between and within DNA sequencing runs (Figure 1).

### Molecular methods

DNA was prepared from frozen sputum as previously described [24]. Briefly, samples were treated with Sputolysin (EMD Chemicals, Gibbstown, NJ, USA) and subjected to mechanical disruption by bead beating before DNA extraction by the MagNA Pure nucleic acid purification platform (Roche Diagnostics Corp., Indianapolis, IN, USA). Total bacterial load was measured by quantitative PCR using the universal primer/probe set of Nadkarni *et al.* [25] targeting the bacterial 16S rRNA gene as previously described [24].

### Virus detection

Samples were selected for viral testing whenever a subject's clinical state changed (that is, from no symptoms to having symptoms or *vice versa*) or at least weekly when clinical state was unchanged for more than 7 days. The FilmArray Respiratory Panel (BioFire Diagnostics, Salt Lake City, UT, USA) was used to test sputum samples for the presence of 17 respiratory viruses [26,27]. Briefly, 1 ml of sputum was liquefied with SnotBuster SL Solution (Copan Diagnostics Inc., Murrieta, GA, USA) by mixing for 30 s at 2,000 rpm. After incubating at room temperature for 15 min, 300 μl of the solution was transferred to sample buffer and inoculated into the FilmArray pouch, which was loaded onto the FilmArray instrument according to the manufacturer's instructions.

### DNA sequencing and community ecological metrics

Bar-coded pyrosequencing of a portion of the 16S rRNA V3-V5 hypervariable region was performed by the Human Genome Sequencing Center at Baylor College of Medicine using protocols developed for the Human Microbiome Project (http://www.hmpdacc.org/resources/tools_protocols.php) as previously described [7], with the following modifications. The software package mothur v1.29 [28] was used to process sequences, which were assigned to OTUs using an average neighbor algorithm with a 0.03 dissimilarity cutoff as previously described [24]. The total number of reads for each community was rarefied to 1,807, the smallest number of reads obtained in the sample set, to control for differences in sequencing depth before alpha (nonparametric Shannon index [29]) and community dissimilarity measures were calculated. BC dissimilarity was used to measure the difference in community structures between paired samples [30]. Dominant OTU was defined as the most abundant OTU in the majority of baseline samples.

### Measure of community stability

Community structure stability was quantified by defining, for each subject, a ‘stability zone’ within which variations in community composition can be explained by stochastic events (stable). Movement of the community outside of this zone is likely due to a perturbation that overrides these stochastic events (unstable) [31]. For each time point, the similarity in community composition was calculated between that time point and the next time point as [1 − Bray-Curtis dissimilarity]. This similarity score, used as a proxy for stability, ranges from 0 to 1. A value of 0 indicates no similarity between communities (implying maximum instability), while a value of 1 indicates no differences between communities (implying perfect stability). We calculated the expected variability in this score due to stochastic processes by randomly permuting counts across the OTU-by-time point matrix for each patient 1,000 times. This generated 1,000 random matrices with neither sample nor temporal selection, while controlling for subject specificity, sampling depth, and global taxon abundance. For each permutation, the stability score [1 − Bray-Curtis dissimilarity] was calculated at all time points. Finally, 95% confidence intervals based on these 1,000 simulated values at each time point was used to create the stability zone.

### Data analysis

Spearman's rank correlation coefficient *P* was calculated to study the associations between community measures, patient metadata, and time. A correlation coefficient *P* ≥ 0.8 was considered a very strong correlation, *P* = 0.60 to 0.79 a strong correlation, *P* = 0.4 to 0.59 a moderate correlation, *P* = 0.20 to 0.39 a weak correlation, and *P* ≤ 0.19 a negligible correlation. HOMOVA [32,33] analysis was used to test whether groups of baseline samples from each subject had equal variances in community structure. BC-based NMDS was used to visualize changes in community composition over time.

## Availability of supporting data

Sequences have been deposited at NCBI SRA (SRA: SRP051730; BioProject: PRNJA271691).

## Additional files

Additional file 1: Table S1. Relative abundance of top OTUs in daily samples. Relative abundance of all OTUs in daily sputum samples from fours subjects. Additional file 2: Figure S1. Diversity in daily samples. Shannon diversity (A) and evenness (B) over time in daily sputum samples from four subjects. Dashed vertical lines indicate transition between baseline and exacerbation. Each plot ends on the day preceding prescription of antibiotics for treatment of exacerbation. Additional file 3: Figure S2. Total 16S rRNA copies per ml of sputum measured by qPCR. Bacterial load is shown during periods of clinical stability (blue) that ended with the onset of exacerbation symptoms (pink). Dashed lines indicate more than one day between samples. Each plot ends on the day preceding prescription of antibiotics for treatment of exacerbation.

## Abbreviations

CF: cystic fibrosis
NMDS: nonmetric multidimensional scaling
BC: Bray Curtis
OTU: operational taxonomic unit
HOMOVA: homogeneity of molecular variance

## References

[1] Rogers GB, Carroll MP, Serisier DJ, Hockey PM, Jones G, Bruce KD (2004). Characterization of bacterial community diversity in cystic fibrosis lung infections by use of 16 s ribosomal DNA terminal restriction fragment length polymorphism profiling. J Clin Microbiol.

[2] Sibley CD, Parkins MD, Rabin HR, Duan K, Norgaard JC, Surette MG (2008). A polymicrobial perspective of pulmonary infections exposes an enigmatic pathogen in cystic fibrosis patients. Proc Natl Acad Sci U S A.

[3] Stressmann FA, Rogers GB, Marsh P, Lilley AK, Daniels TW, Carroll MP (2011). Does bacterial density in cystic fibrosis sputum increase prior to pulmonary exacerbation?. J Cyst Fibros.

[4] Tunney MM, Klem ER, Fodor AA, Gilpin DF, Moriarty TF, McGrath SJ (2011). Use of culture and molecular analysis to determine the effect of antibiotic treatment on microbial community diversity and abundance during exacerbation in patients with cystic fibrosis. Thorax.

[5] Fodor AA, Klem ER, Gilpin DF, Elborn JS, Boucher RC, Tunney MM (2012). The adult cystic fibrosis airway microbiota is stable over time and infection type, and highly resilient to antibiotic treatment of exacerbations. PLoS One.

[6] Stressmann FA, Rogers GB, van der Gast CJ, Marsh P, Vermeer LS, Carroll MP (2012). Long-term cultivation-independent microbial diversity analysis demonstrates that bacterial communities infecting the adult cystic fibrosis lung show stability and resilience. Thorax.

[7] Carmody LA, Zhao J, Schloss PD, Petrosino JF, Murray S, Young VB (2013). Changes in cystic fibrosis airway microbiota at pulmonary exacerbation. Ann Am Thorac Soc.

[8] Daniels TW, Rogers GB, Stressmann FA, van der Gast CJ, Bruce KD, Jones GR (2013). Impact of antibiotic treatment for pulmonary exacerbations on bacterial diversity in cystic fibrosis. J Cyst Fibros.

[9] Price KE, Hampton TH, Gifford AH, Dolben EL, Hogan DA, Morrison HG (2013). Unique microbial communities persist in individual cystic fibrosis patients throughout a clinical exacerbation. Microbiome.

[10] Smith AL, Fiel SB, Mayer-Hamblett N, Ramsey B, Burns JL (2003). Susceptibility testing of *Pseudomonas aeruginosa* isolates and clinical response to parenteral antibiotic administration: lack of association in cystic fibrosis. Chest.

[11] Cunningham S, McColm JR, Mallinson A, Boyd I, Marshall TG (2003). Duration of effect of intravenous antibiotics on spirometry and sputum cytokines in children with cystic fibrosis. Pediatr Pulmonol.

[12] Konstan MW, Morgan WJ, Butler SM, Pasta DJ, Craib ML, Silva SJ (2007). Risk factors for rate of decline in forced expiratory volume in one second in children and adolescents with cystic fibrosis. J Pediatr.

[13] Sanders DB, Hoffman LR, Emerson J, Gibson RL, Rosenfeld M, Redding GJ (2010). Return of FEV1 after pulmonary exacerbation in children with cystic fibrosis. Pediatr Pulmonol.

[14] Erb-Downward JR, Thompson DL, Han MK, Freeman CM, McCloskey L, Schmidt LA (2011). Analysis of the lung microbiome in the "healthy" smoker and in COPD. PLoS One.

[15] Willner D, Haynes MR, Furlan M, Schmieder R, Lim YW, Rainey PB (2012). Spatial distribution of microbial communities in the cystic fibrosis lung. ISME J.

[16] Willner D, Haynes MR, Furlan M, Hanson N, Kirby B, Lim YW (2012). Case studies of the spatial heterogeneity of DNA viruses in the cystic fibrosis lung. Am J Respir Cell Mol Biol.

[17] Brown PS, Pope CE, Marsh RL, Qin X, McNamara S, Gibson R, Burns JL, Deutsch G, Hoffman LR. Directly sampling the lung of a young child with cystic fibrosis reveals diverse microbiota. Ann Am Thorac Soc. 2014 Jul 29. [Epub ahead of print]. 10.1513/AnnalsATS.201311-383OC PMC4214062 25072206

[18] Ding T, Schloss PD (2014). Dynamics and associations of microbial community types across the human body. Nature.

[19] Hoek RA, Paats MS, Pas SD, Bakker M, Hoogsteden HC, Boucher CA (2013). Incidence of viral respiratory pathogens causing exacerbations in adult cystic fibrosis patients. Scand J Infect Dis.

[20] Flight WG, Bright-Thomas RJ, Tilston P, Mutton KJ, Guiver M, Morris J (2013). Incidence and clinical impact of respiratory viruses in adults with cystic fibrosis. Thorax.

[21] Etherington C, Naseer R, Conway SP, Whitaker P, Denton M, Peckham DG (2014). The role of respiratory viruses in adult patients with cystic fibrosis receiving intravenous antibiotics for a pulmonary exacerbation. J Cyst Fibros.

[22] Modi AC, Quittner AL (2003). Validation of a disease-specific measure of health-related quality of life for children with cystic fibrosis. J Pediatr Psychol.

[23] Goss CH, Quittner AL (2007). Patient-reported outcomes in cystic fibrosis. Proc Am Thorac Soc.

[24] Zhao J, Schloss PD, Kalikin LM, Carmody LA, Foster BK, Petrosino JF (2012). Decade-long bacterial community dynamics in cystic fibrosis airways. Proc Natl Acad Sci U S A.

[25] Nadkarni MA, Martin FE, Jacques NA, Hunter N (2002). Determination of bacterial load by real-time pcr using a broad-range (universal) probe and primers set. Microbiology.

[26] Branche AR, Walsh EE, Formica MA, Falsey AR (2014). Detection of respiratory viruses in sputum from adults by use of automated multiplex PCR. J Clin Microbiol.

[27] Ruggiero P, McMillen T, Tang YW, Babady NE (2014). Evaluation of the BioFire FilmArray respiratory panel and the GenMark eSensor respiratory viral panel on lower respiratory tract specimens. J Clin Microbiol.

[28] Schloss PD, Westcott SL, Ryabin T, Hall JR, Hartmann M, Hollister EB (2009). Introducing mothur: open-source, platform-independent, community-supported software for describing and comparing microbial communities. Appl Environ Microbiol.

[29] Chao A, Shen T-J (2003). Nonparametric estimation of Shannon's index of diversity when there are unseen species in sample. Environ Ecol Stat.

[30] Bray JR, Curtis JT (1957). An ordination of the upland forest communities of southern Wisconsin. Ecol Monogr.

[31] Sul WJ, Oliver TA, Ducklow HW, Amaral-Zettler LA, Sogin ML (2013). Marine bacteria exhibit a bipolar distribution. Proc Natl Acad Sci U S A.

[32] Stewart CN, Excoffier L (1996). Assessing population genetic structure and variability with RAPD data: application to *Vaccinium macrocarpon* (American Cranberry). J Evol Biol.

[33] Schloss PD (2008). Evaluating different approaches that test whether microbial communities have the same structure. ISME J.

